# Preoperative prediction of cholangiocyte phenotype hepatocellular carcinoma on contrast-enhanced MRI and the prognostic implication after hepatectomy

**DOI:** 10.1186/s13244-023-01539-x

**Published:** 2023-11-14

**Authors:** Yidi Chen, Jie Chen, Chongtu Yang, Yuanan Wu, Hong Wei, Ting Duan, Zhen Zhang, Liling Long, Hanyu Jiang, Bin Song

**Affiliations:** 1 Department of Radiology, West China Hospital, Sichuan University, Guoxue Road No. 37, Chengdu, 610041 Sichuan China; 2 Big Data Research Center, University of Electronic Science and Technology of China, Chengdu, Sichuan China; 3 Department of Radiology, the First Affiliated Hospital of Guangxi Medical University, Nanning, Guangxi China; 4 Department of Radiology, Sanya People’s Hospital, Sanya, Hainan China

**Keywords:** Hepatocellular carcinoma, Magnetic resonance imaging, Cytokeratin 7, Cytokeratin 19, Cholangiocyte phenotype

## Abstract

**Background:**

Hepatocellular carcinoma (HCC) expressing cytokeratin (CK) 7 or CK19 has a cholangiocyte phenotype that stimulates HCC proliferation, metastasis, and sorafenib therapy resistance This study aims to noninvasively predict cholangiocyte phenotype-positive HCC and assess its prognosis after hepatectomy.

**Methods:**

Between January 2010 and May 2022, preoperative contrast-enhanced MRI was performed on consecutive patients who underwent hepatectomy and had pathologically confirmed solitary HCC. Two abdominal radiologists separately assessed the MRI features. A predictive model for cholangiocyte phenotype HCC was created using logistic regression analysis and five-fold cross-validation. A receiver operating characteristic curve was used to calculate the model performance. Kaplan–Meier and log-rank methods were used to evaluate survival outcomes.

**Results:**

In total, 334 patients were included in this retrospective study. Four contrast-enhanced MRI features, including “rim arterial phase hyperenhancement” (OR = 5.9, 95% confidence interval [CI]: 2.9–12.0, 10 points), “nodule in nodule architecture” (OR = 3.5, 95% CI: 2.1–5.9, 7 points), “non-smooth tumor margin” (OR = 1.6, 95% CI: 0.8–2.9, 3 points), and “non-peripheral washout” (OR = 0.6, 95% CI: 0.3–1.0, − 3 points), were assigned to the cholangiocyte phenotype HCC prediction model. The area under the curves for the training and independent validation set were 0.76 and 0.73, respectively. Patients with model-predicted cholangiocyte phenotype HCC demonstrated lower rates of recurrence-free survival (RFS) and overall survival (OS) after hepatectomy, with an estimated median RFS and OS of 926 vs. 1565 days (*p* < 0.001) and 1504 vs. 2960 days (*p* < 0.001), respectively.

**Conclusions:**

Contrast-enhanced MRI features can be used to predict cholangiocyte phenotype-positive HCC. Patients with pathologically confirmed or MRI model-predicted cholangiocyte phenotype HCC have a worse prognosis after hepatectomy.

**Critical relevance statement:**

Four contrast-enhanced MRI features were significantly associated with cholangiocyte phenotype HCC and a worse prognosis following hepatectomy; these features may assist in predicting prognosis after surgery and improve personalized treatment decision-making.

**Key points:**

• Four contrast-enhanced MRI features were significantly associated with cholangiocyte phenotype HCC.

• A noninvasive cholangiocyte phenotype HCC predictive model was established based on MRI features.

• Patients with cholangiocyte phenotype HCC demonstrated a worse prognosis following hepatic resection.

**Graphical Abstract:**

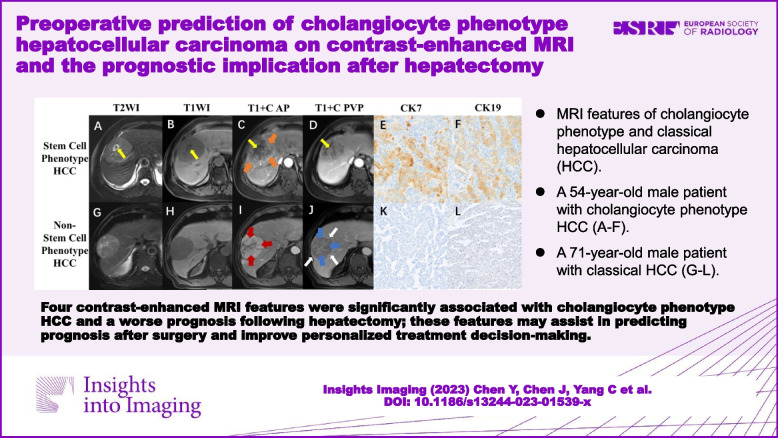

**Supplementary Information:**

The online version contains supplementary material available at 10.1186/s13244-023-01539-x.

## Background

Liver cancer is the sixth most commonly diagnosed cancer and causes the third highest number of cancer-related deaths worldwide [1]. Hepatocellular carcinoma (HCC) represents 75–90% of all primary liver cancer cases [2]. Complex pathological phenotypes and tumor heterogeneity are the main causes of poor prognosis in patients with HCC. HCC expressing cytokeratin (CK) 7 or CK19 are considered cholangiocyte phenotypes with highly aggressive behavior that can stimulate HCC proliferation, metastasis, and sorafenib therapy resistance [3, 4]. Cholangiocyte phenotype-positive HCCs show morphological signatures of HCC (arising from hepatocytes rather than cholangiocytes), but simultaneously express phenotypical features of hepatocytes and cholangiocytes. Both CK7 and CK19 are cholangiocyte-specific markers expressed in HCC that may be mediated by cancer stem cells (CSCs) [5, 6].

These features of cholangiocyte phenotype HCC imply that diagnosis requires pathological examination and immunohistochemical staining for hepatocyte- and cholangiocyte-specific markers. Invasive procedures can be performed to obtain specimens; however, these biopsy specimens are susceptible to sampling errors. Therefore, biopsy is not frequently performed as part of the preoperative workup.

Contrast-enhanced magnetic resonance imaging (MRI) to assess the expression of CK7 and CK19 in HCC has been investigated. Gadoxetic acid-enhanced MRI (EOB-MRI) is a valuable technique for evaluating CK19 expression in HCC [7]. Furthermore, EOB-MRI-based histogram analysis is valuable for predicting HCC based on CK19 expression [8], and radiomics analysis of MRI is highly useful for assessing the expression of CK7 and CK19, as well as other histopathological aspects of HCC [9–12]. In addition, CK19 expression in HCC has been evaluated using various quantitative MRI diffusion models [13]. Despite promising results, the limited clinical adoption of quantitative techniques and unsatisfactory interpretability of radiomics have hampered their application in routine clinical practice.

HCC with CK7- or CK19-positve expression have similar biological features, but previous studies have discussed their imaging and prognostic characteristics separately. In this study, we proposed a cholangiocyte phenotype of HCC (i.e., positive expression of CK7 and/or CK19), which may be more effective in identifying this specific HCC. To the best of our knowledge, a noninvasive and simple prediction model for identifying the cholangiocyte phenotype of HCC has not been proposed in any published studies.

This study aimed to develop a noninvasive prediction model for cholangiocyte phenotype-positive HCC based on readily accessible preoperative clinical features and MRI findings and to validate its performance in predicting patient outcomes following curative-intent liver resection.

## Methods

### Subjects

This retrospective study was conducted at a single center and was approved by the Ethics Committee of West China Hospital, Sichuan University (Approval No. 2022–651). The requirement for written informed consent was waived due to the retrospective nature of this study.

**Fig. 1:**
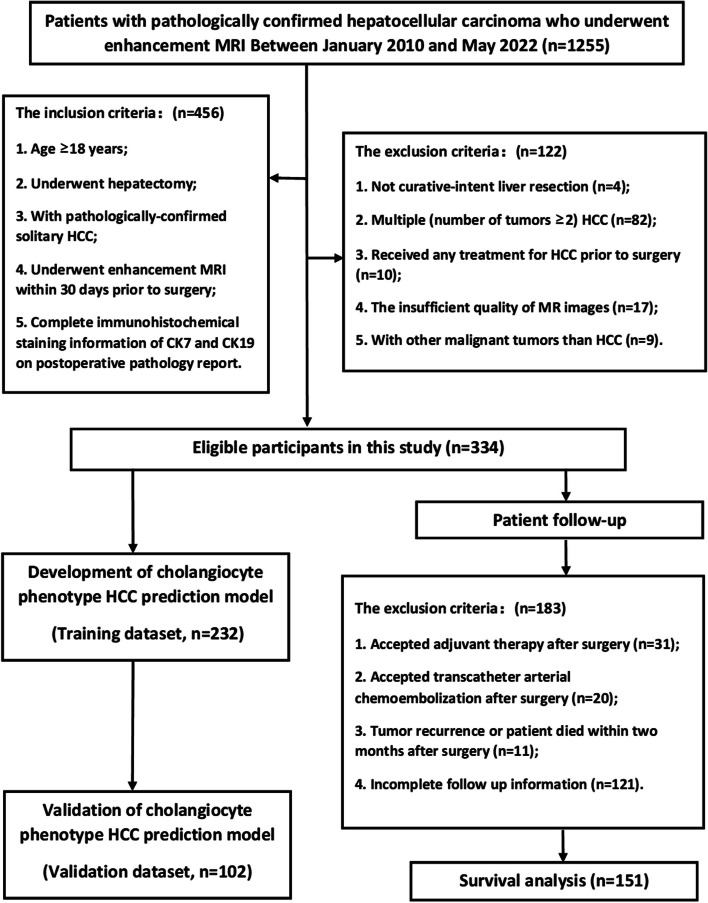
Flowchart of the retrospective study cohort. A total of 334 patients diagnosed with solitary hepatocellular carcinoma were included in this research

From January 2010 to May 2022, 334 eligible patients (283 males) with a mean age of 53.0 ± 11.6 years were included in this study (Fig. 1), who met the following inclusion criteria: (a) no less than 18 years of age, (b) underwent curative hepatic resection, (c) had pathologically confirmed HCC, (d) underwent contrast-enhanced MRI within 30 days before surgery, and (e) underwent complete immunohistochemical staining for CK7 and CK19 which was included on the postoperative pathology report. The exclusion criteria were as follows: (a) received non-curative liver resection; (b) had multiple HCC (HCCs equal to or more than two); (c) received any antitumor treatment for HCC prior to surgery; (d) had insufficient MR imaging quality (e.g., severe artifact); and (e) presence of malignant tumors other than HCC (for example, combined hepatocellular-cholangiocarcinoma [cHCC-CCA] and sarcomatoid carcinoma, cHCC-CCA contains areas of both typical HCC and typical iCCA, the former having any/all of the possible cytological and architectural features of HCCs and the latter distinctly being an adenocarcinoma with malignant glands, usually lying within a dense stromal background). Details of patient inclusion and exclusion criteria are presented in Fig. 1.

Baseline clinical information, including patient demographics, causes of liver disease, and key laboratory test results (alpha-fetoprotein [AFP] and carbohydrate antigen 199 [CA199]) within 14 days of surgery, were obtained from electronic medical records.

### MRI acquisition and analysis

Four 3.0-T MR scanners (Discovery 750, SIGNA™ Architect and SIGNA™ Premier, GE Healthcare; and MAGNETOM Skyra, Siemens Healthineers) and one 1.5-T MRI scanner (uMR588, United Imaging Healthcare) were used to acquire MR images. The sequences employed T2-weighted imaging, diffusion-weighted imaging, T1-weighted in-phase, and opposed-phase imaging, as well as T1-weighted dynamic contrast-enhanced imaging using gadopentetic acid dimeglumine or gadoxetic acid disodium (Primovist®, Bayer Pharma AG). Supplementary A1 and Table S1 provide detailed information regarding the MRI techniques.

Two radiologists with 8 and 6 years of experience in liver MR, respectively, independently assessed all MR scans. Although the reviewers were aware that all patients had HCC, they remained blinded to other clinical, histopathological, and follow-up information. In cases where there was a discrepancy in image interpretation, a third radiologist with over 20 years of experience in liver MR assessed the image to provide a resolution.

A total of 24 pre-operative MRI features were assessed. These features encompass those related to the underlying liver disease (e.g., radiologically evident cirrhosis) and other prognostic features (e.g., intratumoral artery, tumor growth subtype, non-smooth tumor margin, and peritumoral hepatobiliary phase hypointensity) of HCC. Descriptions of the assessed features are summarized in Supplementary Table (S2).

### Histopathology and immunohistochemistry

Data on tumor location, number, size, Edmondson-Steiner differentiation grade, immunohistochemical expression of CK7 and CK19, hepatocyte paraffin antigen 1 (HepPar-1), glypican-3 (GPC-3), glutamine synthetase (GS), and microvascular invasion were collected from pathology reports. The expression of CK7, CK19, HepPar-1, GPC-3, and GS was classified as negative or positive. All histopathological examinations were conducted by two pathologists (with over 5 and 10 years of experience in liver pathology) who were blinded to the clinical and imaging information. HCC with the cholangiocyte phenotype was pathologically diagnosed if all of the following criteria were fulfilled: (I) microscopic morphological features of HCC; (II) positive expression of HepPar-1, GPC-3, or GS in tumor cells; and (III) positive expression of CK7 and/or CK19 in tumor cells (≥ 15%) [5, 6, 14].

### Patient follow-up

After surgery, patients were followed up at 1 month, 2 months, and then every 3 months for the first 2 years. Subsequently, follow-ups were performed every 6 months. During each follow-up, serum AFP levels were measured, and contrast-enhanced ultrasound, CT, or MR imaging was performed. Additionally, tumor recurrence was confirmed by imaging or pathological examination during the follow-up period. The administration of adjuvant therapy (e.g., systemic therapy and transcatheter arterial chemoembolization) prior to recurrence had been documented. Recurrence-free survival (RFS) was defined as the duration from the date of surgery to the occurrence of tumor recurrence or the last follow-up date (May 1, 2022), whichever occurred first. Patients who died from causes unrelated to tumor recurrence were censored without an event when calculating RFS. Overall survival (OS) was defined as the duration from the date of surgery to the date of death from any cause or the last follow-up date, whichever occurred first.

### Development and validation of the cholangiocyte phenotype HCC prediction model

A predictive model for cholangiocyte phenotype HCC was developed and validated. According to the chronological order of MRI examinations, those patients included in this study were divided into a model training dataset (232 patients) and an external validation dataset (102 patients) in a 7:3 ratio.

In the training dataset, univariate logistic regression analyses were conducted to identify clinicoradiological features associated with the cholangiocyte phenotype of HCC. Continuous variables were converted into categorical or dichotomized variables based on normality ranges or clinical relevance to enhance their clinical applicability. Multicollinearity was assessed using the variance inflation factor. All independent predictors with *p*-values < 0.1 in the univariate analyses were included in the multivariate logistic regression model, which utilized the backward stepwise method and fivefold cross-validation to create an “internal validation” dataset. Patient age, sex, and hepatitis B virus infection status (infected vs. non-infected) were controlled for in the model. Akaike Information Criterion was used to obtain the most parsimonious feature combination. Therefore, these features were selected because their combination allowed the lowest Akaike Information Criterion among all feature combinations. However, this approach did not correspond to all *p* < 0.05 [15, 16]. A scoring system was developed using the predictors identified in the multivariate regression analysis to estimate the probability of the cholangiocyte phenotype in HCC. The optimal threshold of the scoring system was determined using the receiver operating characteristic (ROC) curve analysis and Youden's index.

A ROC curve was used to compute the area under the curve (AUC), sensitivity, specificity, positive predictive value (PPV), negative predictive value (NPV), and accuracy of the discriminative performance of the model. Calibration curves were plotted to assess the calibration of the model using the Hosmer–Lemeshow test. Furthermore, decision curve analysis was conducted to evaluate the clinical utility of the model by quantifying the net benefits at various threshold probabilities.

### Statistical analysis

The Shapiro–Wilk test was used to assess normal distributions. Differences in continuous variables were analyzed using either the independent samples *t*-test or the Mann–Whitney *U* test. Categorical variables were evaluated using the chi-square test or Fisher’s exact test.

Cohen’s κ values or weighted κ values were used to evaluate the inter-rater agreement between the two reviewers in the MRI analysis.

The Kaplan–Meier technique and log-rank test were used to evaluate survival outcomes. Patients were excluded from the survival analysis if they had received systemic therapy (*n* = 31) or transcatheter arterial chemoembolization (*n* = 20) before recurrence, if tumor recurrence or death occurred within 2 months after surgery (*n* = 11), and if follow-up data were incomplete (*n* = 121).

All statistical analyses were conducted using the R project for statistical computation (version 4.0.5). Statistical significance was set at *p* < 0.05.

## Results

### Study population

Among the enrolled patients, 269 (80.5%) had hepatitis B, two (0.6%) had hepatitis C, 21 (6.3%) had both hepatitis B and C, and 42 (12.6%) had other causes of liver disease, such as alcoholic liver disease, non-alcoholic fatty liver disease, autoimmune liver disease, or cholestatic cirrhosis.

A total of 138 (41.3%) patients had cholangiocyte phenotype-positive HCC and 196 (58.7%) had classical HCC. In the cholangiocyte phenotype and classical group, the mean patient age was 50.8 ± 11.5 years and 54.5 ± 11.4 years, respectively (*p* = 0.004); the median serum AFP was 19.9 (range: 1.1–2112.0) ng/mL and 26.6 (range: 1.3–1210.0) ng/mL, respectively *(p* = 0.344); CA 19–9 was 15.2 (range 1.0–1000.0) U/mL and 17.4 (range 1.0–1000.0) U/mL, respectively *(p* = 0.375) (Table 1). No significant differences were observed in baseline clinical features between the training and validation datasets (Supplementary Table S3) (all* p* > 0.05).

**Table 1:** The clinical characteristics of patients with HCC

Variables	All patients (*n* = 334)	Cholangiocyte phenotype HCC (*n* = 138)	Classical HCC (*n* = 196)	*p* value
Age^a^ (years)	53.0 ± 11.6	50.8 ± 11.5	54.5 ± 11.4	0.004
Sex (*n*, %)				0.359
Male	283 (84.7%)	120 (87.0%)	163 (83.2%)	
Female	51 (15.3%)	18 (13.0%)	33 (16.8%)	
AFP ^b^ (ng/mL)	23.1 (1.1–2112.0)	19.9 (1.1–2112.0)	26.6 (1.3–1210.0)	0.344
CA199^b^ (U/mL)	16.4 (1.0–1000.0)	15.2 (1.0–1000.0)	17.4 (1.0–1000.0)	0.375
CEA^b^ (ng/mL)	2.2 (1.0–16.0)	2.1 (1.0–7.0)	2.3 (1.0–16.0)	0.076
TBIL^b^ (μmol/L)	14.8 (5.2–537.7)	14.6 (5.2–537.7)	14.9 (5.3–499.2)	0.395
DBIL^b^ (μmol/L)	5.2 (1.0–424.0)	5.1 (1.5–418.8)	5.3 (1.0–424.0)	0.112
IBIL^b^ (μmol/L)	9.5 (3.0–118.9)	9.3 (3.0–118.9)	9.6 (3.5–75.2)	0.625
ALT^b^ (U/L)	34.0 (10.0–753.0)	32.0 (10.0–753.0)	34.0 (11.0–607.0)	0.770
AST^b^ (U/L)	32.0 (14.0–845.0)	31.0 (16.0–845.0)	33.5 (14.0–450.0)	0.972
ALB^b^ (g/L)	44.5 (23.1–147.0)	44.8 (31.2–147.0)	44.4 (23.1–55.3)	0.193
PLT^b^ (10^9^/L)	125.0 (25.0–470.0)	136.0 (25.0–285.0)	120.0 (26.0–470.0)	0.060
Cause of liver disease				0.524
HBV	269 (80.5%)	115 (83.4%)	154 (78.6%)	
HCV	2 (0.6%)	1 (0.7%)	1 (0.5%)	
HBV + HCV	21 (6.3%)	9 (6.5%)	12 (6.1%)	
OTHER^c^	42 (12.6%)	13 (9.4%)	29 (14.8%)	
BCLC stage				0.834
0	70 (21.0%)	29 (21.0%)	41 (20.9%)	
A	226 (67.7%)	95 (68.9%)	131 (66.9%)	
C	38 (11.3%)	14 (10.1%)	24 (12.2%)	

*AFP* alpha-fetoprotein, *CEA* carcinoma embryonic antigen, *TBIL* total bilirubin, *DBIL* direct bilirubin, *IBIL* indirect bilirubin, *ALT* alanine transaminase, *AST* aspartate aminotransferase, *ALB* serum albumin, *PLT* platelet count, *PT* prothrombin time, *HBV* hepatitis B virus, *HCV* hepatitis C virus, *BCLC stage* Barcelona clinic liver cancer stage

^a^described as mean (SD)

^b^described as median (range)

The OTHER^c^ causes of liver disease included alcoholic liver disease, non-alcoholic fatty liver disease, autoimmune liver disease, and cholestatic cirrhosis

### Correlations between MRI features and cholangiocyte phenotype HCC

The number of lesions with LI-RADS categories 4, 5, and M was 12 (8.7%), 105 (76.1%), and 21 (15.2%), respectively, for patients with cholangiocyte phenotype-positive HCC, and 19 (9.7%), 161 (82.1%), and 16 (8.2%) for classical HCC, respectively (*p* = 0.129). In the cholangiocyte phenotype and classical group, the number of lesions presenting “rim arterial phase hyperenhancement (APHE)” were 45 (32.6%) and 14 (7.1%), respectively (*p* < 0.001); the number of lesions presenting “nonperipheral washout” were 95 (68.8%) and 160 (81.6%), respectively (*p* = 0.009); the number of lesions presenting “non-smooth tumor margin” were 116 (84.1%) and 128 (65.3%), respectively (*p* < 0.001); and the number of lesions presenting “nodule in nodule” were 81 (58.7%) and 60 (30.6%), respectively (*p* < 0.001); all the other analyzed MRI features are detailed in Table 2.

**Table 2:** MRI features and consistency analysis between the two HCC groups with cholangiocyte phenotype and non-cholangiocyte phenotype

Variables	Cholangiocyte phenotype HCC (*n* = 138)	Classical HCC (*n* = 196)	*p* value	Kappa	*p* value
**Size** (cm)	3.4 (1.0–17.1)	3.2 (0.8–20.0)	0.522	-	-
**Tumor margin**			< 0.001	0.514	< 0.001
Smooth	22 (15.9%)	68 (34.7%)			
Non-smooth	116 (84.1%)	128 (65.3%)			
**Tumor growth subtype**			0.163*	0.475	< 0.001
Single nodular type	50 (36.2%)	95 (48.5%)			
Single nodule type with Extra-nodular growth	77 (55.8%)	88 (44.9%)			
Contiguous multinodular type	4 (2.9%)	5 (2.5%)			
Infiltrative type	7 (5.1%)	8 (4.1%)			
**Marked diffusion restriction**			0.244	0.570	< 0.001
Presence	28 (20.3%)	30 (15.3%)			
Absence	110 (79.7%)	166 (84.7%)			
**Marked T2 hyperintense**			0.179	0.494	< 0.001
Presence	9 (6.5%)	6 (3.1%)			
Absence	129 (93.5%)	190 (96.9%)			
**Fat in mass more than liver**			0.356	0.418	< 0.001
Presence	46 (33.3%)	76 (38.8%)			
Absence	92 (66.7%)	120 (61.2%)			
**Fat sparing in solid mass**			0.801	0.481	< 0.001
Presence	8 (5.8%)	9 (4.6%)			
Absence	130 (94.2%)	187 (95.4%)			
**Non-rim APHE**			< 0.001	0.613	< 0.001
Presence	95 (58.8%)	179 (91.3%)			
Absence	43 (31.2%)	17 (8.7%)			
**Rim APHE**			< 0.001	0.574	< 0.001
Presence	45 (32.6%)	14 (7.1%)			
Absence	93 (67.4%)	182 (92.9%)			
**Internal artery**			0.807	0.601	< 0.001
Presence	39 (28.3%)	59 (30.1%)			
Absence	99 (71.17%)	137 (69.9%)			
**Corona enhancement**			0.220	0.547	< 0.001
Presence	70 (50.7%)	85 (43.4%)			
Absence	68 (49.3%)	111 (56.6%)			
**Nonperipheral washout**			0.009	0.494	< 0.001
Presence	95 (68.8%)	160 (81.6%)			
Absence	43 (31.2%)	36 (18.4%)			
**Peripheral washout**			0.004*	0.300	< 0.001
Presence	8 (5.8%)	1 (0.5%)			
Absence	130 (94.2%)	195 (99.5%)			
**Delayed central enhancement**			< 0.001	0.389	< 0.001
Presence	19 (13.8%)	6 (3.1%)			
Absence	119 (86.2%)	190 (96.9%)			
**PVP peritumoral hypo-enhancement**			0.997	0.613	< 0.001
Presence	31 (22.5%)	44 (22.4%)			
Absence	107 (77.5%)	152 (77.6%)			
**Complete capsule**			< 0.001	0.538	< 0.001
Presence	18 (13.0%)	60 (30.6%)			
Absence	120 (87.0%)	136 (69.4%)			
**Blood products in mass**			0.801	0.683	< 0.001
Presence	37 (26.8%)	50 (25.5%)			
Absence	101 (73.2%)	146 (74.5%)			
**Nodule in nodule**			< 0.001	0.412	< 0.001
Presence	81 (58.7%)	60 (30.6%)			
Absence	57 (41.3%)	136 (69.4%)			
**Mosaic architecture**			0.029	0.552	< 0.001
Presence	51 (37.0%)	50 (25.5%)			
Absence	87 (63.0%)	146 (74.5%)			
**Infiltrative appearance**			0.741	0.632	< 0.001
Presence	19 (13.8%)	24 (12.2%)			
Absence	119 (86.2%)	172 (87.8%)			
**Necrosis or severe ischemia**			0.543	0.775	< 0.001
Presence	38 (27.5%)	61 (31.1%)			
Absence	100 (72.5%)	135 (68.9%)			
**Tumor in vein**			0.822	0.786	< 0.001
Presence	8 (5.8%)	13 (6.6%)			
Absence	130 (94.2%)	183 (93.4%)			
**LI_RADS**			0.129	0.412	< 0.001
4	12 (8.7%)	19 (9.7%)			
5	105 (76.1%)	161 (82.1%)			
M	21 (15.2%)	16 (8.2%)			

*APHE* arterial phase hyperenhancement, *PVP* portal vein phase

^*^Fisher’s exact test

### Development of the cholangiocyte phenotype HCC prediction model

**Fig. 2:**
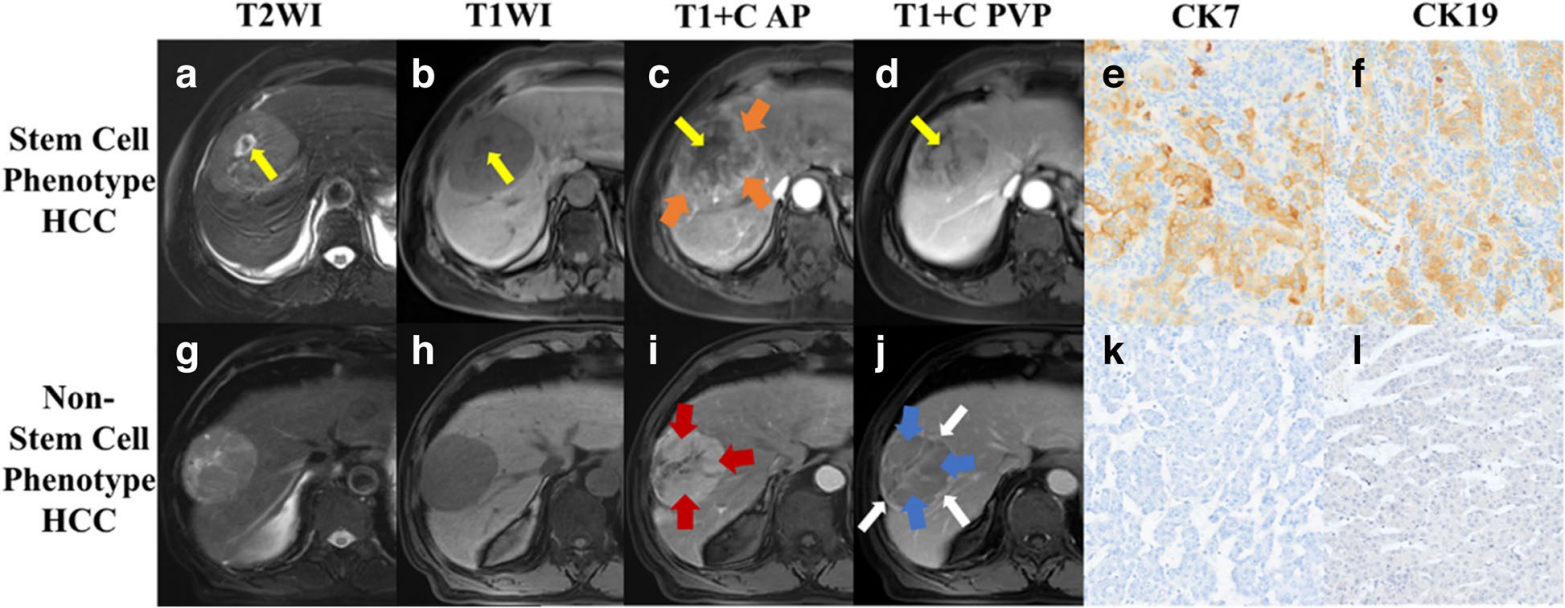
MRI features of cholangiocyte phenotype and classical hepatocellular carcinoma (HCC). A 54-year-old male patient with cholangiocyte phenotype HCC (**a**–**f**), and a 71-year-old male patient with classical HCC (**g**–**l**). T2WI showed hyperintense lesions (**a**, **g**), pre-contrast T1WI showed hypointense lesions (**b**, **h**), and “nodule in nodule architecture” was observed in the cholangiocyte phenotype HCC (**a**–**d**, yellow arrow). Arterial phase images showed “rim arterial phase hyperenhancement (APHE)” (**c**, orange arrow) and “non-rim APHE” (**i**, red arrow). Portal venous phase images showed “peripheral washout” with nodular delayed enhancement (**d**, yellow arrow) and “nonperipheral washout” (**j**, blue arrow) and smooth margin with enhanced capsule (**j**, white arrow); immunohistochemical staining revealed the CK7 (**e**, **k**, × 100) and CK19 (**f**, **l**, × 100) positive (**e**, **f**) and negative (**k**, **l**) expressions, respectively

Four MRI features were associated with the cholangiocyte phenotype HCC and were used to construct the scoring system (RNNN score) based on multivariable logistic regression analysis and the fivefold cross-validation, including “rim APHE” (odds ratio [OR] = 5.9, 95% confidence interval [CI]: 2.9–12.0, corresponding to 10 points in the scoring system), “nodule in nodule architecture” (OR = 3.5, 95% CI: 2.1–5.9, corresponding to 7 points in the scoring system), non-smooth tumor margin (OR = 1.6, 95% CI: 0.8–2.9, corresponding to 3 points in the scoring system), and “non-peripheral washout” (OR = 0.6, 95% CI: 0.3–1.0, corresponding to − 3 points in the scoring system) (Fig. 2, Table 3).

**Table 3:** The selected MRI features to predict the cholangiocyte phenotype HCC

	Univariable			Multivariable		
Variables	Coefficient	*p* value	OR (95%CI)	Coefficient	*p* value	OR (95%CI)
AFP (> 100 ng/mL vs. ≤ 100 ng/mL)	− 0.476	0.043	0.621 (0.392–0.985)	-	-	-
CA199 (> 30 U/mL vs. ≤ 30 U/mL)	− 0.175	0.531	0.839 (0.485–1.452)	-	-	-
Size (> 3.0 cm vs. ≤ 3.0 cm)	0.181	0.419	1.198 (0.773–1.856)	-	-	-
Tumor margin	1.030	< 0.001	2.801 (1.628–4.818)	0.443	0.155	1.557 (0.845–2.871)
Tumor growth subtype (single nodular vs. other)	0.504	0.027	1.655 (1.060–2.586)	-	-	-
Marked diffusion restriction	0.343	0.238	1.408 (0.798–2.487)	-	-	-
Marked T2 hyperintense	0.793	0.142	2.209 (0.768–6.357)	-	-	-
Fat in mass more than liver	− 0.236	0.309	0.789 (0.500–1.245)	-	-	-
Fat sparing in solid mass	0.246	0.622	1.279 (0.481–3.401)	-	-	-
Non-rim APHE	− 1.561	< 0.001	0.210 (0.114–0.388)	-	-	-
Rim APHE	1.839	< 0.001	6.290 (3.285–12.046)	1.769	< 0.001	5.866 (2.868–11.998)
Internal artery	− 0.089	0.716	0.915 (0.566–1.478)	-	-	-
Corona enhancement	0.296	0.185	1.344 (0.868–2.082)	-	-	-
Nonperipheral washout	− 0.699	0.007	0.497 (0.298–0.828)	-0.573	0.058	0.564 (0.311–1.021)
Peripheral washout	2.485	0.020	12.00 (1.483–97.084)	-	-	-
Delayed central enhancement	1.621	0.001	5.056 (1.963–13.021)	-	-	-
PVP peritumoral hypo-enhancement	0.001	0.997	1.001 (0.594–1.687)	-	-	-
Complete capsule	− 1.079	< 0.001	0.340 (0.190–0.608)	-	-	-
Blood products in mass	0.067	0.790	1.070 (0.652–1.755)	-	-	-
Nodule in nodule	1.170	< 0.001	3.221 (2.043–5.077)	1.263	< 0.001	3.537 (2.118–5.906)
Mosaic architecture	0.538	0.026	1.712 (1.068–2.744)	-	-	-
Infiltrative appearance	0.135	0.682	1.144 (0.600–2.182)	-	-	-
Necrosis or severe ischemia	− 0.173	0.480	0.841 (0.520–1.360)	-	-	-
Tumor in vein	− 0.144	0.757	0.866 (0.349–2.150)	-	-	-
LI-RADS M^#^	0.703	0.046	2.019 (1.012–4.029)	-	-	-

“Single nodular vs. other,” the other included “single nodule type with extranodular growth,” “contiguous multinodular type,” and “infiltrative type”

*AFP* alpha-fetoprotein, *CEA* carcinoma embryonic antigen, *APHE* arterial phase hyperenhancement, *OR* odds ratio

^#^LI-RADS M vs. LI-RADS 4 or 5

RNNN score = 10 × “rim APHE” (presence = 1, absence = 0) + 7 × “nodule in nodule architecture” (presence = 1, absence = 0) + 3 × “non-smooth tumor margin” (presence = 1, absence = 0) − 3 × “non-peripheral washout” (presence = 1, absence = 0).

Following Youden’s index, we calculated the optimal threshold of the RNNN scoring system as 5.5 points. Patients with a total score of ≥ 5.5 points were categorized as having a high risk of cholangiocyte phenotype HCC.

In this study cohort, the inter-rater agreement was good or moderate for “rim APHE” (*κ* = 0.613, 95% CI: 0.521–0.691), “nodule in nodule architecture” (*κ* = 0.412, 95% CI: 0.360–0.466), “non-smooth tumor margin” (*κ* = 0.514, 95% CI: 0.458–0.564), and “non-peripheral washout” (*κ* = 0.494, 95% CI: 0.431–0.547). Table 2 shows the remaining inter-rater agreements for the imaging features.

### Validation of the cholangiocyte phenotype HCC prediction model

**Fig. 3:**
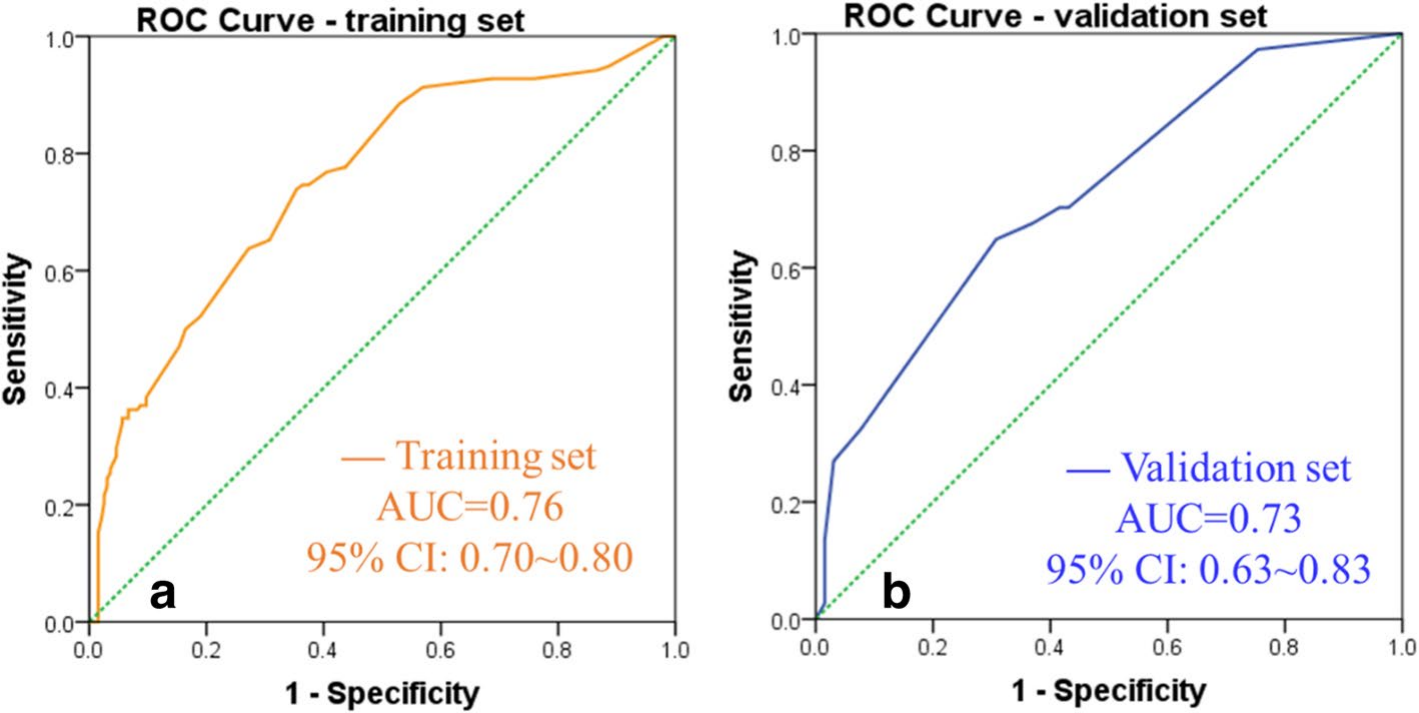
Receiver operating curves of the cholangiocyte phenotype-positive hepatocellular carcinoma predictive model. The prediction model’s area under the curve (AUC) was 0.76 (95% CI: 0.70–0.80) and 0.73 (95% CI: 063–0.83) (*p* < 0.05) for the training and independent validation datasets, respectively

The RNNN model’s AUCs for training and independent validation datasets were 0.76 (95% CI: 0.695–0.800) and 0.73 (95% CI: 0631–0.826), respectively (Fig. 3, Table 4). Based on the threshold of 5.5 points, for the independent validation dataset, the sensitivity and specificity for the prediction model were 65% (95% CI: 64.2%–79.7%) and 70% (95% CI: 56.6%–80.1%), respectively.

**Table 4:** The performance of predictive model for cholangiocyte phenotype HCC

	Training set (*n* = 232)	Independent validation set (*n* = 102)
AUC and 95% CI	0.76 (0.70–0.80)	0.73 (0.63–0.83)
Sensitivity and 95% CI	72.5% (64.2–79.7%)	64.9% (47.5–79.8%)
Specificity and 95% CI	68.4% (61.4–74.8%)	70.0% (56.6–80.1%)
PPV and 95% CI	61.7% (53.8–69.2%)	54.5% (38.8–69.6%)
NPV and 95% CI	77.9% (71.0–83.9%)	77.6% (64.7–87.5%)
ACC and 95% CI	70.1% (64.8–74.9%)	67.6% (57.7–76.6%)

*AUC* area under the curve, *95% CI* 95% confidence interval, *PPV* positive predictive value, *NPV* negative predictive value, *ACC* accuracy

**Fig. 4:**
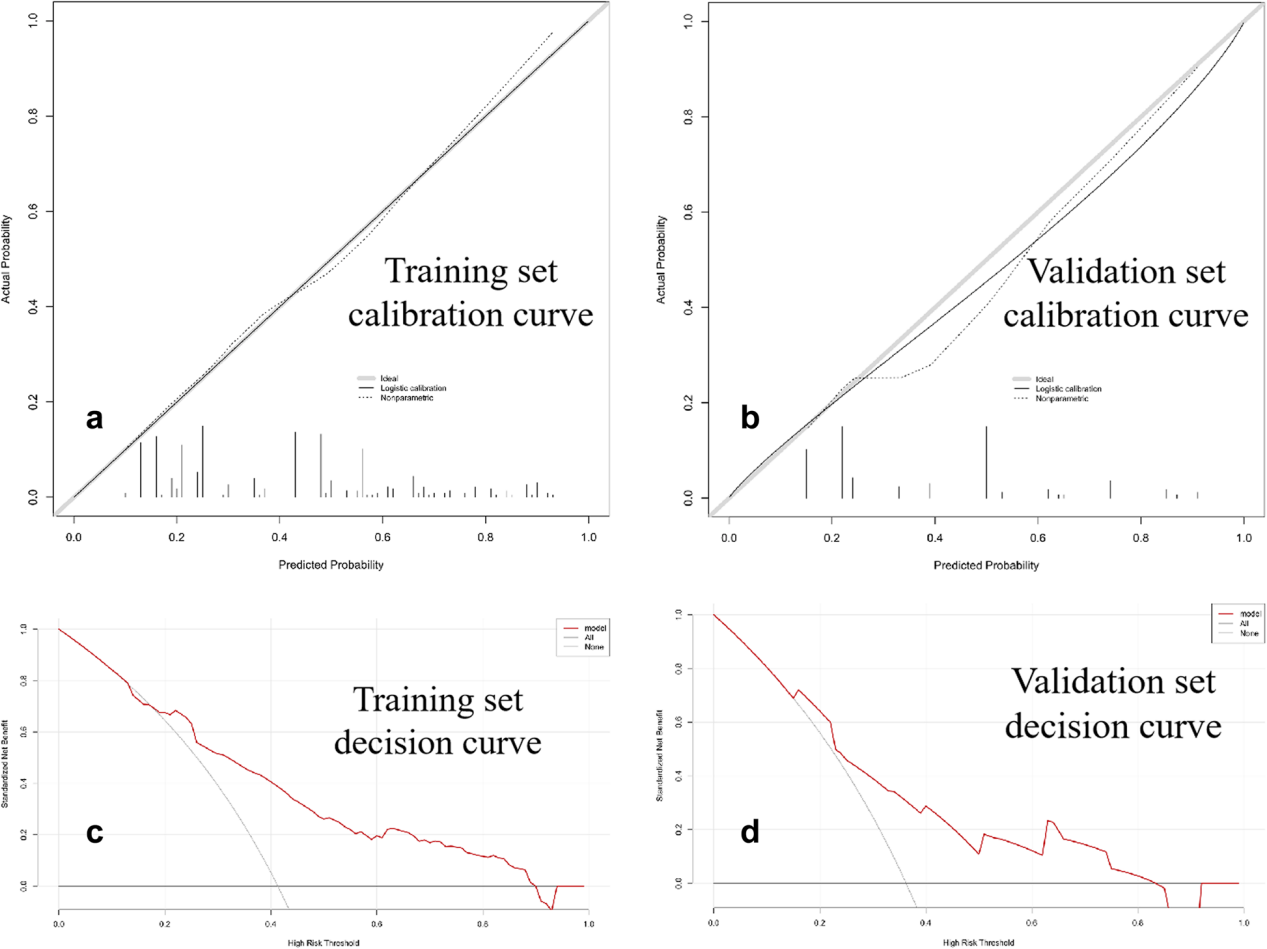
Calibration (**a**, **b**) and decision curve (**c**, **d**) to predict cholangiocyte phenotype-positive hepatocellular carcinoma (HCC). The calibration curves exhibited satisfactory concordance between the predicted and observed probabilities of cholangiocyte phenotype HCC in both the training (**a**) and independent validation (**b**) datasets. The decision curve analysis of the prediction model was performed for the training (**c**) and independent validation (**d**) datasets. The net benefit, calculated based on true positives and false positives, was plotted on the *Y*-axis, while the *X*-axis represented the probability threshold. The curve of the predictive model demonstrated favorable benefits

The calibration curves indicated strong concordance between the predicted and observed probabilities of cholangiocyte phenotype HCC in both the training and validation datasets. Decision curve analysis provided further validation, confirming that our predictive model performed well in accurately assessing the preoperative risk of cholangiocyte phenotyped HCC (Fig. 4).

### Survival analysis

**Fig. 5:**
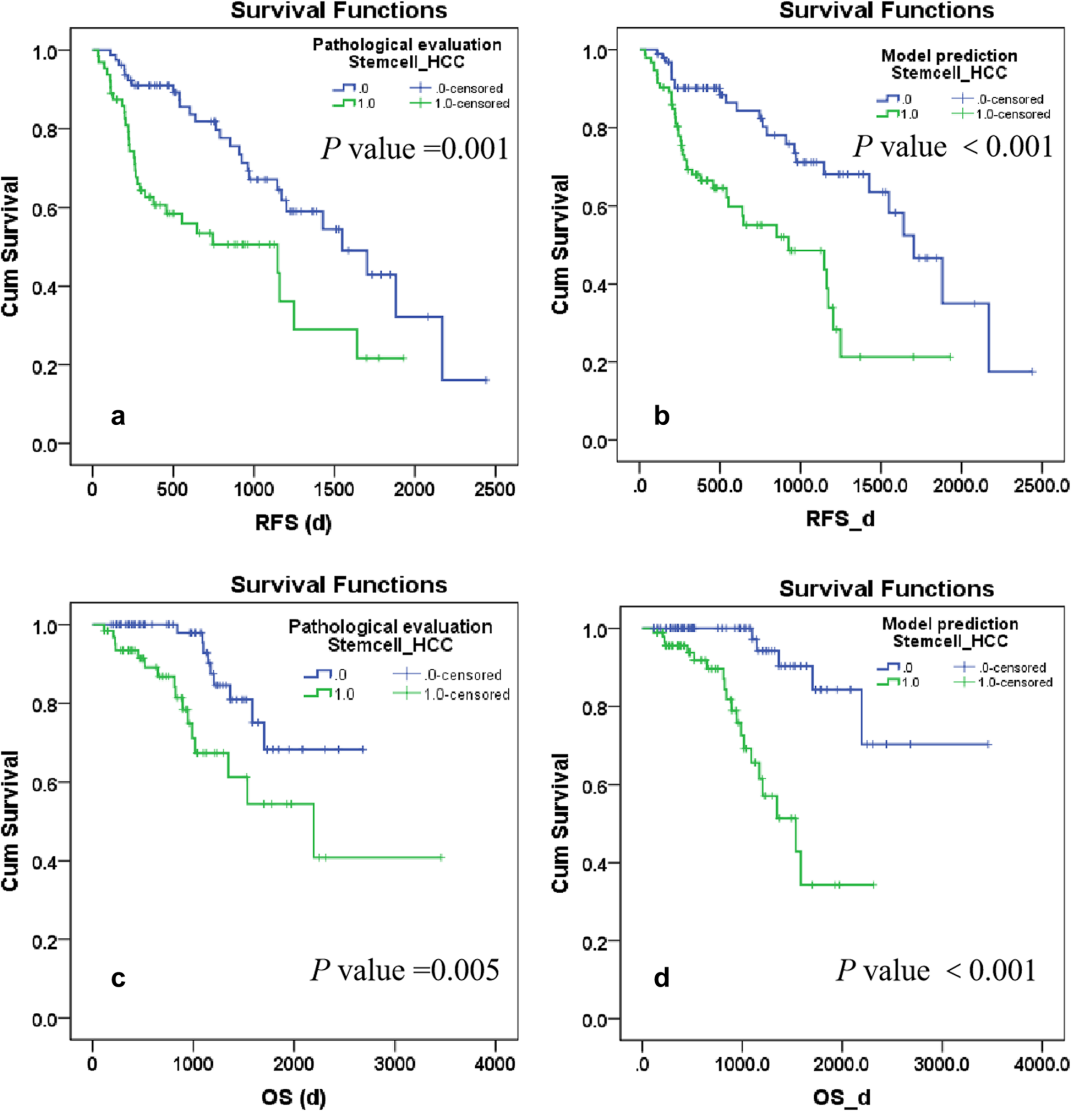
The Kaplan–Meier plots for postoperative prognosis. Pathologically confirmed (**a**, **c**) and model-predicted (**b**, **d**) cholangiocyte phenotype-positive hepatocellular carcinoma (stemcell_HCC) showed the stratifying efficacy in postoperative recurrence-free survival (RFS) (**a**, **b**) and overall survival (OS) (**c**, **d**)

A total of 151 eligible patients were followed up for a median duration of 759 days (range: 112–3460 days); 28 patients died and 59 patients had tumor recurrence. The median RFS and OS were 541 days and 894 days, respectively. Patients with cholangiocyte phenotype HCC had a poorer prognosis after hepatic resection. Significant disparities in RFS and OS were observed between patients with pathologically confirmed cholangiocyte phenotype HCC and those without this phenotype. The estimated median RFS was 933 days vs. 1490 days (*p* = 0.001), while the median OS was 2126 days vs. 2260 days (*p* = 0.005). Furthermore, significant differences were observed in RFS and OS between patients with model-predicted cholangiocyte phenotype and non-cholangiocyte phenotype HCC. The estimated median RFS was 926 vs. 1565 days (*p* < 0.001), while the median OS was 1504 vs. 2960 days (*p* < 0.001) (Fig. 5).

## Discussion

Through research involving patients who underwent curative hepatectomy for solitary hepatocellular carcinoma (HCC), we developed and validated a simple and non-invasive risk score to predict cholangiocyte phenotype HCC based on four MRI features (“rim APHE,” “nodule in nodule architecture,” “non-smooth tumor margin,” and “non-peripheral washout”). The model demonstrated an AUC of 0.73, good calibration, and substantial decision-making effectiveness in predicting the cholangiocyte phenotype of HCC. Postoperative RFS and OS were worse in patients with cholangiocyte phenotype HCC. Therefore, the effectiveness of the predictive model for categorizing postoperative survival was determined.

MRI features can predict HCC subclasses. Seo-Youn et al. [7] demonstrated that irregular margins, arterial phase rim enhancement, and a lower tumor-to-liver signal intensity ratio in hepatobiliary phase imaging could potentially aid in predicting CK19-positive HCC. Chen et al. [8] revealed irregular tumor margins, targetoid appearance, and absence of mosaic architecture were noteworthy indicators of HCC exhibiting the progenitor phenotype HCC. Our results demonstrated that when the MRI features of “rim APHE” and “nodule-in-nodule architecture” are detected, whether in combination with other MRI features or not, it is highly suggestive of the cholangiocyte phenotype HCC.

The subtype of targetoid morphology known as “Rim APHE” is likely indicative of peripheral hypercellularity, central stromal fibrosis, or ischemia. This feature is most commonly observed in HCC with atypical phenotypes, such as intrahepatic cholangiocarcinoma (iCCA) and cHCC-CCA [17]. Previous investigations [18–20] have documented the association between “Rim APHE” and indicators of minor differentiation, infiltrative growth, presence of microvascular invasion, and rapid growth accompanied by central necrosis. Additionally, several studies [7, 8] have shown that progenitor phenotype HCC frequently presents with a targetoid appearance and arterial rim enhancement. Similarly, our study demonstrated “Rim APHE” is an effective predictor for HCC with the cholangiocyte phenotype; this may suggest that cholangiocyte and progenitor cell phenotype HCC share some common pathological and physiological mechanisms.

The term “Nodule-in-nodule architecture” describes the occurrence of a smaller inner nodule within a larger outer nodule. The inner nodule often displays traits of advanced HCC resulting from the clonal expansion of cells along the hepatocarcinogenesis pathway [21]. Hence, HCC with a “nodule-in-nodule architecture” may indicate the presence of various tumor stem cells at different developmental stages. Stepwise evolution of cancer was observed in HCC with a nodule-in-nodule appearance through multiregional whole-genome sequencing analyses. In addition, within an immortalized cellular population, specific tumor cells may acquire multiple genetic aberrations associated with different oncogenic pathways. This process leads to the transformation of slow-growing tumor cells into aggressive malignant cells [22]. Our study largely confirmed this theory, as the results demonstrated that the MRI feature of “nodule-in-nodule architecture” is an independent risk factor for cholangiocyte phenotype HCC.

“Non-peripheral washout” is a frequent imaging feature of HCC, with a specificity of greater than 90% for typical HCC [23, 24]. Mature HCC tissues receive less portal flow than the background parenchyma, which has a washout appearance [25]. Choi et al. [7] reported that the “arterial enhancement with washout” feature is present more commonly in CK19-negative HCC than CK 19-positive HCC (93.1% Vs. 78.9%). This finding is consistent with our results. In our study cohort, the presence of “non-peripheral washout” was more commonly observed in the cholangiocyte phenotype than in other types of HCC (81.6% vs. 78.9%). Consequently, this feature was incorporated into the cholangiocyte phenotype HCC prediction model as a protective factor (OR = 0.6, corresponding to − 3 points in the scoring system).

During the study follow-up, 25 patients died and 59 patients had tumor recurrence. Kaplan–Meier survival curves showed poorer survival in patients with cholangiocyte phenotype HCC. Moreover, our results demonstrated that the MRI model had better prediction efficiency for RFS and OS. This may be because MRI features comprehensively reflect the biological characteristics of a single HCC, as reported in a previous study by Rhee et al. [18]. The study illustrated that HCC with the image feature of “irregular rim arterial phase enhancement” displayed aggressive histopathologic traits such as increased stemness and hypoxic and fibrotic tumor microenvironments, and had unfavorable disease-free survival outcomes. Similarly, according to the findings of Hyo-Jin et al. [20], the presence of “rim APHE” correlated with poor overall survival rates and a higher incidence of extrahepatic metastasis among patients with HCC. Our results highlight that the MRI features included in the predictive model may serve as non-invasive imaging biomarkers for aggressive HCC.

## Limitations

This study has several limitations. First, as this was a single-center study, the lack of external validation may have weakened the generalization of our predictive model. Second, in our study, the rate of cholangiocyte phenotype HCC (41.3%) was higher than that in previous reports; the possible reason is that in this study, the presence of either CK7 or CK19 positive expression in HCC is sufficient to define it as cholangiocyte phenotype HCC. Third, a previous study [26] revealed that cancer stem cells could activate and maintain DNA damage repair signaling after treatment with sorafenib and that some medicines may enhance therapeutic efficiency by suppressing DNA damage repair signaling. However, we did not directly evaluate this therapeutic effect in cholangiocyte phenotyped HCC.

## Conclusions

We developed and validated an efficient and non-invasive cholangiocyte phenotype HCC predictive scoring system based on four MRI features. We additionally demonstrated that patients with pathologically confirmed or MRI model-predicted cholangiocyte phenotype HCC have a worse prognosis after hepatectomy. Our findings may help improve personalized treatment decisions.

### Supplementary Information

**Additional file 1: Supplementary A1.** **Supplementary Table S1.** MRI sequences and parameters in our institution. **Supplementary Table S2.** Definitions of the evaluated MR imaging features. **Supplementary Table S3.** baseline clinical and MRI features among the training and validation datasets.

## Data Availability

The datasets utilized during the present study can be obtained from the corresponding author upon a reasonable request.

## Abbreviations

APHE: Arterial phase hyperenhancement
CK19: Cytokeratin 19
CK7: Cytokeratin 7
CSCs: Cancer stem cells
GPC-3: Glypican-3
GS: Glutamine synthetase
HCC: Hepatocellular carcinoma
HepPar-1: Hepatocyte paraffin antigen 1
LI-RADS: The Liver Imaging Reporting and Data System
MRI: Magnetic resonance imaging
NPV: Negative predictive value
OS: Overall survival
PPV: Positive predictive value
RFS: Recurrence-free survival
