# Supplementary material for: Preoperative prediction of cholangiocyte phenotype hepatocellular carcinoma on contrast-enhanced MRI and the prognostic implication after hepatectomy
Source: Insights Imaging. 2023 Nov 14;14:190. doi: 10.1186/s13244-023-01539-x (PMC10645671; doi:10.1186/s13244-023-01539-x)
Supplement: Supplementary file 1 — Additional file 1: Supplementary A1. Supplementary Table S1. MRI sequences and parameters in our institution. Supplementary Table S2. Definitions of the evaluated MR imaging features. Supplementary Table S3. baseline clinical and MRI features among the training and validation datasets. [file 13244_2023_1539_MOESM1_ESM.docx]

**Preoperative Prediction of Cholangiocyte Phenotype Hepatocellular Carcinoma on Contrast-enhanced MRI and the Prognostic Implication After Hepatectomy**

**ELECTRONIC SUPPLEMENTARY MATERIAL**

**Supplementary A1**

**MRI** **technique**

MRI examinations were performed with four 3.0-T systems (MAGNETOM Skyra, Siemens Healthineers; Discovery MR 750, GE Healthcare; SIGNA™ Architect, GE Healthcare; and SIGNA™ Premier, GE Healthcare) and a 1.5-T system (uMR588, United Imaging Healthcare). The MRI sequences included T2-weighted two-dimensional fast spin echo imaging, diffusion-weighted imaging (b values: 0, 50, 500, 800, 1000, and 1200 s/mm^2^ [Siemens MAGNETOM Skyra]; 0, 200, 800, and 1000 s/mm^2^ [GE Discovery MR 750]; 0, 50, and 800 s/mm^2^ [GE SIGNA™ Architect 3.0 Tesla]; 50 and 1000 s/mm^2^ [GE SIGNA™ Premier 3.0 Tesla]; and 0, 50, and 1000 s/mm^2^ [uMR588 1.5 Tesla]) with apparent diffusion coefficient (ADC) maps, T1-weighted dual gradient-echo in- and opposed-phase imaging, and dynamic T1- weighted three-dimensional gradient-echo imaging before and after injection of gadoxetic acid disodium (Primovist®, Bayer Pharma AG) in the late arterial phase, portal venous phase (60 s), transitional phase (3 minutes), and hepatobiliary phase (20 minutes). The arterial phase images were determined either by the acquisition triggered 7 s after the arrival of the contrast bolus in the celiac trunk or by a multiple arterial phase (MAP) imaging technique. Specifically, the MAP images were acquired with an 18 s breath hold 20 s following the contrast agent injection and further reconstructed with a temporal resolution of 3 s. For dynamic imaging, the contrast agent was injected at a rate of 1-2 ml/s for a total dose of 0.025 mmol/kg body weight, followed by 20-30 ml of 0.9% saline flush. Details of the MRI sequences and parameters are shown in **Table S1.**

**Table S1** MRI sequences and parameters in our institution

| Sequence | T1-weighted IP and OP imaging | Dynamic T1-weighted 3D GRE | T2-weighted 2D FSE | Diffusion-weighted imaging^†^ |
| --- | --- | --- | --- | --- |
| Siemens MAGNETOM Skyra (18-channel body array coil) | | | | |
| Repetition time (ms) | 81 | 3.95 | 2160 | 5600 |
| Echo time (ms) | 2.72/1.4 | 1.92 | 100 | 68 |
| Flip angle (°) | 70 | 9 | 160 | 90 |
| Section thickness (mm) | 6 | 2.5 | 6 | 6 |
| Spacing (mm) | 1.8 | - | 1.8 | 1.8 |
| Matrix size | 352×286 | 352×256 | 320×288 | 100×76 |
| Field of view (mm^2^) | 400×325 | 400×296 | 433×433 | 380×289 |
| Acquisition time (s) | 24 | 14 | 36 | 233 |
| Fat suppression | No | Yes | Yes | Yes |
| GE Discovery MR 750 (16-channel phased-array torsor coil) | | | | |
| Repetition time (ms) | 150 | 4.1 | 6315 | 9230 |
| Echo time (ms) | 2.5/1.3 | 1.9 | 78 | Minimum |
| Flip angle (°) | 70 | 15 | 111 | 90 |
| Section thickness (mm) | 6 | 2 | 6 | 6 |
| Spacing (mm) | 2 | - | 2 | 2 |
| Matrix size | 288×192 | 512×512 | 288×244 | 128 × 128 |
| Field of view (mm^2^) | 420×420 | 380× 300 | 360×280 | 360× 380 |
| Acquisition time (s) | 31 | 15 | RG | RG |
| Fat suppression | No | Yes | Yes | Yes |
| GE SIGNA™ Architect 3.0 Tesla (30-channel body anterior coil) | | | | |
| Repetition time (ms) | 233.8 | 3.9 | 2400 | 5000 |
| Echo time (ms) | 2.3/1.1 | 1.7 | 85 | Minimum |
| Flip angle (°) | 55 | 15 | 111 | 90 |
| Section thickness (mm) | 7 | 3 | 7 | 7 |
| Spacing (mm) | 2 | - | 2 | 2 |
| Matrix size | 160×288 | 320×240 | 320×192 | 160×128 |
| Field of view (mm^2^) | 380×323 | 380×380 | 380×304 | 380×342 |
| Acquisition time (s) | 18 | 15 | 34 | RG |
| Fat suppression | No | Yes | Yes | Yes |
| GE SIGNA™ Premier 3.0 Tesla (30-channel body anterior coil) | | | | |
| Repetition time (ms) | 146.8 | 3.2 | 2200 | 5000 |
| Echo time (ms) | 2.3/1.1 | 1.4 | 85 | Minimum |
| Flip angle (°) | 55 | 15 | 111 | 90 |
| Section thickness (mm) | 7 | 2.4 | 7 | 7 |
| Spacing (mm) | 2 | - | 2 | 2 |
| Matrix size | 320×192 | 320×240 | 320×224 | 120 × 240 |
| Field of view (mm^2^) | 342×380 | 380× 380 | 304×380 | 380× 380 |
| Acquisition time (s) | 16 | 15 | 47 | RG |
| Fat suppression | No | Yes | Yes | Yes |
| uMR588 1.5 Tesla (6-channel body anterior coil) | | | | |
| Repetition time (ms) | 117.6 | 4.2 | 2600 | 3350 |
| Echo time (ms) | 2.27 | 1.88 | 99.2 | 77 |
| Flip angle (°) | 60 | 10 | 90 | 90 |
| Section thickness (mm) | 6.5 | 2.5 | 6.5 | 6.5 |
| Spacing (mm) | 1.3 | - | 1.5 | 10 |
| Matrix size | 256×174 | 256×154 | 256×168 | 128×92 |
| Field of view (mm^2^) | 320×400 | 255×400 | 427×320 | 320×400 |
| Acquisition time (s) | 29 | 13 | 39 | RG |
| Fat suppression | No | Yes | Yes | Yes |

MRI, magnetic resonance imaging; IP, in-phase; OP, opposed-phase; 3D, three-dimensional; GRE, gradient recall echo; 2D, two-dimensional; FSE, fast spin-echo; RG, respiratory gating.

^†^Images were acquired under free breath.

**Table S2** Definitions of the evaluated MR imaging features

| MRI feature | Definition |
| --- | --- |
| Tumor Size | Largest outer-edge-to-outer-edge dimension of a liver observation |
| Tumor margin | Non-smooth tumor margin: presence of non-nodular tumors with irregular contour that had budding portion at the periphery |
| Tumor growth subtype | (1) Single nodular type;(2) single nodule type with extra-nodular growth; (3) contiguous multinodular type; (4) infiltrative type. |
| Marked diffusion restriction | Increased signal intensity of the liver observation at diffusion-weighted imaging in relative to the spleen, not solely attributable to T2-weighted imaging shine-through effect |
| Marked T2 hyperintensity | Signal intensity of the liver observation on T2 weighed-imaging markedly higher than liver and similar to bile ducts and other fluid-filled structures |
| Fat in mass, more than adjacent liver | Excess fat within a mass, in whole or in part, relative to adjacent liver |
| Fat sparing in solid mass | Relative paucity of fat in solid mass relative to steatotic liver OR in inner nodule relative to steatotic outer nodule |
| Nonrim arterial phase hyperenhancement (APHE) | Nonrim-like enhancement of the liver observation in arterial phase unequivocally greater in whole or in part than liver |
| Rim arterial phase hyperenhancement (APHE) | Presence of arterial phase enhancement most pronounced in observation periphery |
| Internal artery | Presence of discrete arterial enhancement within the tumor |
| Corona enhancement | Periobservational enhancement in late arterial phase or early portal venous phase attributable to venous drainage from tumor |
| Nonperipheral "washout" | Nonperipheral visually assessed temporal reduction in enhancement of the liver observation in whole or in part relative to composite liver tissue from earlier to later phase resulting in hypoenhancement in the extracellular phase |
| Peripheral "washout" | Presence of apparent washout most pronounced in observation periphery |
| Delayed central enhancement | Central area of progressive postarterial phase enhancement |
| PVP peritumoral hypo-enhancement | Presence of wedge-shaped or flame-like hypointense area adjacent to the tumor border on portal venous phase images |
| Tumor capsule | -Complete: Presence of non-disrupted "capsule" in all imaging planes;  -Incomplete: Presence of disrupted "capsule" in all imaging planes |
| Blood products in mass | Intralesional or perilesional hemorrhage in the absence of biopsy, trauma or intervention |
| Nodule-in-nodule architecture | Presence of smaller inner nodule within and having different imaging features than larger outer nodule |
| Mosaic architecture | Presence of randomly distributed internal nodules or compartments, usually with different imaging features |
| Infiltrative appearance | Liver observation with non-circumscribed margin (indistinct transition) |
| Necrosis or severe ischemia | Presence of unequivocal intralesional necrosis or severe ischemia |
| Tumor in vein | Presence of unequivocal enhancing soft tissue in vein |
| LI-RADS | Liver Imaging Reporting and Data System |

HCC, hepatocellular carcinoma; PVP, portal venous phase; AP, arterial phase; IP, in-phase; OP, opposed-phase; DWI, diffusion-weighted imaging; T2WI, T2-weighted imaging; T1WI, T1-weighted imaging; LI-RADS/LR, Liver Imaging Reporting and Data System.

**References**

1. CT/MRI Liver Imaging Reporting and Data System version 2018 (2018) Available via [https://www.acr.org/Clinical-Resources/Reporting-and-Data-Systems/LI-RADS/CT-MRI-LI-RADS-v2018. Accessed 25 Oct 2021](https://www.acr.org/Clinical-Resources/Reporting-and-Data-Systems/LI-RADS/CT-MRI-LI-RADS-v2018.%20Accessed%2025%20Oct%202021)
2. Blachar A, Federle MP, Sosna J (2009) Liver lesions with hepatic capsular retraction. Semin Ultrasound CT MR 30:426-435
3. Ji GW, Zhu FP, Xu Q et al (2020) Radiomic features at contrast-enhanced CT predict recurrence in early stage hepatocellular carcinoma: a multi-institutional study. Radiology 294:568-579
4. Renzulli M, Brocchi S, Cucchetti A et al (2016) Can current preoperative imaging be used to detect microvascular invasion of hepatocellular carcinoma? Radiology 279:432-442
5. Lee S, Kim SH, Lee JE, Sinn DH, Park CK (2017) Preoperative gadoxetic acid-enhanced MRI for predicting microvascular invasion in patients with single hepatocellular carcinoma. J Hepatol 67:526-534
6. Lei Z, Li J, Wu D et al (2016) Nomogram for preoperative estimation of microvascular invasion risk in hepatitis B virus-related hepatocellular carcinoma within the Milan criteria. JAMA Surg 151:356-363

**Table S3.** baseline clinical and MRI features among the training and validation datasets

| **Variables** | **Training dataset**  **(n=232)** | **Validation dataset**  **(n=102)** | ***P* value** |
| --- | --- | --- | --- |
| **Age** (year) | 54.8±11.2 | 55.6±11.8 | 0.874 |
| **Sex** (n, %) |  |  | 0.509 |
| Male | 199 (85.8%) | 84 (82.4%) |  |
| Female | 33 (14.2%) | 18 (17.6%) |  |
| **AFP** (ng/mL) | 26.6 (1.4~2112.0) | 14.2 (1.1~1210.0) | 0.078 |
| **CA199** (U/ml) | 18.1 (1.0~1000.0) | 13.2 (2.0~175.0) | 0.070 |
| **CEA** (ng/ml) | 2.2 (1.0~16.0) | 2.1 (1.0~9.0) | 0.598 |
| **TBIL** (μmol/L) | 14.8 (5.2~537.7) | 14.6 (5.3~42.2) | 0.932 |
| **DBIL** (μmol/L) | 5.3 (1.5~424.0) | 5.0 (1.0~52.5) | 0.125 |
| **IBIL** (μmol/L) | 9.4 (3.0~118.9) | 9.6 (3.6~47.6) | 0.656 |
| **ALT** (U/L) | 35.0 (10.0~753.0) | 31.0 (10.0~278.0) | 0.360 |
| **AST** (U/L) | 34.0 (16.0~845.0) | 30.0 (14.0~174.0) | 0.094 |
| **ALB** (g/L) | 44.5 (23.1~58.5) | 44.4 (32.9~147.0) | 0.123 |
| **PLT** (10^9^/L) | 123.5 (25.0~470.0) | 126.0 (31.0~349.0) | 0.655 |
| **BCLC stage** |  |  | 0.062 |
| 0 | 49 (21.1%) | 21 (20.6%) |  |
| A | 150 (64.7%) | 76 (74.5%) |  |
| C | 33 (14.2%) | 5 (4.9%) |  |
| **Stem cell HCC** |  |  | 0.229 |
| Presence | 101 (43.5%) | 37 (36.3%) |  |
| Absence | 131 (56.5%) | 65 (63.7%) |  |
| **Size** (cm) | 4.5±3.3 | 4.1±2.8 | 0.147 |
| **Tumor margin** |  |  | 0.143 |
| Smooth | 57 (24.6%) | 33 (32.4%) |  |
| Non-smooth | 175 (75.4%) | 69 (67.6%) |  |
| **Tumor growth subtype** |  |  | 0.061 |
| Single nodular type | 98 (42.2%) | 47 (46.1%) |  |
| Single nodule type with Extra-nodular growth | 116 (50.0%) | 49 (48.0%) |  |
| Contiguous multinodular type | 4 (1.7%) | 5 (4.9%) |  |
| Infiltrative type | 14 (6.0%) | 1 (1.0%) |  |
| **Marked diffusion restriction** |  |  |  |
| Presence | 35 (15.1%) | 23 (22.5%) | 0.068 |
| Absence | 197 (84.9%) | 79 (77.5%) |  |
| **Marked T2 hyperintense** |  |  | 0.567 |
| Presence | 9 (3.9%) | 6 (5.9%) |  |
| Absence | 223 (96.1%) | 96 (94.1%) |  |
| **Fat in mass more than liver** |  |  | 0.389 |
| Presence | 81 (34.9%) | 41 (40.2%) |  |
| Absence | 151 (65.1%) | 61 (59.8%) |  |
| **Fat sparing in solid mass** |  |  | 1.000 |
| Presence | 12 (5.2%) | 5 (4.9%) |  |
| Absence | 220 (94.8%) | 97 (95.1%) |  |
| **Non-rim APHE** |  |  | 1.000 |
| Presence | 190 (81.9%) | 84 (82.4%) |  |
| Absence | 42 (18.1%) | 18 (17.6%) |  |
| **Rim APHE** |  |  | 0.540 |
| Presence | 43 (18.5%) | 16 (15.7%) |  |
| Absence | 189 (81.5%) | 86 (84.3%) |  |
| **Internal artery** |  |  | 1.000 |
| Presence | 68 (29.3%) | 30 (29.4%) |  |
| Absence | 164 (70.7%) | 72 (70.6%) |  |
| **Corona enhancement** |  |  | 0.341 |
| Presence | 112 (48.3%) | 43 (42.2%) |  |
| Absence | 120 (51.7%) | 59 (57.8%) |  |
| **Nonperipheral washout** |  |  | 1.000 |
| Presence | 177 (76.3%) | 78 (76.5%) |  |
| Absence | 55 (23.7%) | 24 (23.5%) |  |
| **Peripheral washout** |  |  | 1.000 |
| Presence | 6 (2.6%) | 3 (2.9%) |  |
| Absence | 226 (97.4%) | 99 (97.1%) |  |
| **Delayed central enhancement** |  |  | 1.000 |
| Presence | 17 (7.3%) | 8 (7.8%) |  |
| Absence | 215 (92.7%) | 94 (92.2%) |  |
| **PVP peritumoral hypo-enhancement** |  |  | 0.777 |
| Presence | 51 (22.0%) | 24 (23.5%) |  |
| Absence | 181 (78.0%) | 78 (76.5%) |  |
| **Complete capsule** |  |  | 0.161 |
| Presence | 49 (21.1%) | 29 (28.4%) |  |
| Absence | 183 (78.9%) | 73 (71.6%) |  |
| **Blood products in mass** |  |  | 0.787 |
| Presence | 59 (25.4%) | 28 (27.5%) |  |
| Absence | 173 (74.6%) | 74 (72.5%) |  |
| **Nodule in nodule** |  |  | 0.339 |
| Presence | 102 (44.0%) | 39 (38.2%) |  |
| Absence | 130 (56.0%) | 63 (61.8%) |  |
| **Mosaic architecture** |  |  | 0.093 |
| Presence | 77 (33.2%) | 24 (23.5%) |  |
| Absence | 155 (66.8%) | 78 (76.5%) |  |
| **Infiltrative appearance** |  |  | 0.293 |
| Presence | 33 (14.2%) | 10 (9.8%) |  |
| Absence | 199 (85.8%) | 92 (90.2%) |  |
| **Necrosis or severe ischemia** |  |  | 0.796 |
| Presence | 70 (30.2%) | 29 (28.4%) |  |
| Absence | 162 (69.8%) | 73 (71.6%) |  |
| **Tumor in vein** |  |  | 0.140 |
| Presence | 18 (7.8%) | 3 (2.9%) |  |
| Absence | 214 (92.2%) | 99 (97.1%) |  |
| **LI_RADS** |  |  | 0.973 |
| 4 | 22 (9.5%) | 9 (8.8%) |  |
| 5 | 184 (79.3%) | 82 (80.4%) |  |
| M | 26 (11.2%) | 11 (10.8%) |  |

Data are expressed as n (%).

AFP, alpha-fetoprotein; CEA, carcinoma embryonic antigen; TBIL, total bilirubin; DBIL, direct bilirubin; IBIL, indirect bilirubin; ALT, alanine transaminase; AST, aspartate aminotransferase; ALB, serum albumin; PLT, platelet count; PT, prothrombin time; HBV, hepatitis B virus; HCV, hepatitis C virus; BCLC stage, Barcelona clinic liver cancer stage.

LI-RADS/LR, Liver Imaging Reporting and Data System; APHE, arterial phase hyperenhancement.
